# Characteristics of constrained turbulent transport in flux-driven toroidal plasmas

**DOI:** 10.1098/rsta.2021.0231

**Published:** 2023-02-20

**Authors:** Y. Kishimoto, K. Imadera, A. Ishizawa, W. Wang, J. Q. Li

**Affiliations:** ^1^ Gradulate School of Energy Science, Kyoto University, Uji, Kyoto 611-0011, Japan; ^2^ Non-linear and non-equilibrium Plasma Science Research UNIT, Center for the promotion of Interdisciplinary Education and Research, Kyoto University, Uji, Kyoto 611-0011, Japan; ^3^ Southwestern Institute of Physics, Chengdu 610041,People's Republic of China

**Keywords:** magnetically confined fusion plasmas, tubulent transport, non-diffusive transport, global flux-driven simulation, avalanche dynamics, global bursts

## Abstract

We study the dynamics of turbulence transport subject to a constraint on the profile formation and relaxation, dominated by the ion temperature gradient modes, within the framework of adiabatic electron response using a flux-driven global gyro-kinetic toroidal code, GKNET. We observe exponentially constrained profiles, with two different scale lengths, that are spatially constant in each region in higher input power regimes. The profiles are smoothly connected in the knee region located at 1/2−2/3 of the minor radius, outside which the gradient is steepened and shows a weak confinement improvement. Based on the probability density function analysis of heat flux eddies, the power law demonstrates a dependence on the eddy size *S*, as $P∼S^{-\alpha }$, which distinguishes events into diffusive and non-diffusive parts including the validation of quasi-linear hypotheses. Radially localized avalanches and global bursts, which exhibit different spatial scales, play central roles in giving rise to constrained profiles on an equal footing. It is also found that the E×B *shear layers* are initiated by the global bursts, which evolve downward on a slow time scale across the knee region and play a role in adjusting the profile by increasing the gradient.

This article is part of a discussion meeting issue ‘H-mode transition and pedestal studies in fusion plasmas’.

## 1. Introduction

**Figure 1.**
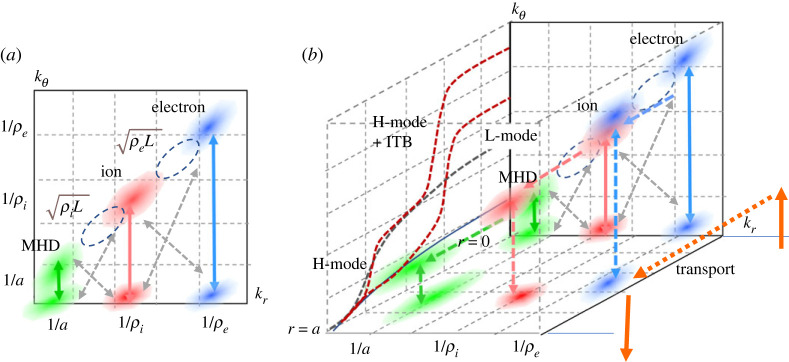
(*a*) Distribution of fluctuations in the $\left(k_{r},k_{\theta }\right)$ plane including those due to linear toroidal coupling and nonlinear driven zonal mode. Possible couplings between different scales are also shown. (*b*) A radial dimension is introduced to (*a*) to represent a system sustained by external source and sink. The feature of radial convection is illustrated together with the possible plasma pressure profile for L-mode, H-mode and an internal transport barrier. (Online version in colour.)

Magnetically confined plasmas, to which energy is supplied externally in a restricted volume such that the power density inevitably increases, are subject to various constraints in structure and dynamics, including magnetic topology [1–3]. One of the constraints is *profile consistency* initially pointed out by Coppi [4], but widely referred to as *profile stiffnes*s [5–12]. This is of specific importance since it directly reflects the global energy confinement scaling which determines the plasma performance, while current physics-based understanding is not developed enough. Due to the constraints on the profile, which may result from various kinds of fluctuation and associated dynamics discussed in figure 1, incorporated with boundary layers connecting to the outer region, e.g. the scrape-off layer and edge pedestal, the global function form of the profile is determined intrinsically, while only the scale length characterizing the spatial extent can be changed during the evolution.

Such fluctuations not only originate from the primary linear free energy source shown in figure 1*a*, but also from secondary and tertiary modes induced from the primary fluctuation through nonlinear processes as shown in figure 1*b*; they are key in regulating the stability, confinement and hence fusion performance. Examples of various zonal modes are zonal flows, their magnetic counterpart, zonal fields [13–16], geodesic acoustic modes [17,18] and generalized Kelvin–Helmholtz modes [19]. Their fluctuations cause complex structures and dynamics not only in configuration space, e.g. turbulent spreading [20–23], avalanches and bursts [24–29], fine-scale pressure corrugations, referred to as staircase [30–36], etc. but also in velocity space, e.g. flattening and vortex formation in phase space via wave-particle interaction [37,38]. These are categorized as mesoscopic transport events, referred to as *non-local* and *non-diffusive* transport, where a flux-gradient relation based on Fick's law and then quasi-linear ansatz are poorly applied. This feature is shown in figure 1*b* by adding the radial dimension to figure 1*a*.

These fluctuations cause a lower confinement state universally observed in experiments with auxiliary heating known as L-mode. Temperature and pressure profiles are hardly changed subject to a constraint over the global plasma scale *L*, referred to as *profile stiffness*. Some experiments show that the inverse scale lengths tend to be approximately constant over a finite radial region, so that exponential profiles were established [5–9,12]. The relation between global confinements and such constrained temperature profiles is discussed based on a critical gradient model by defining a stiffness factor. Paradoxical ingredients, e.g. stiffness, emerge, although key transport-related parameters, such as values of *q* and $\hat{s}$, $T_{e}/T_{i}$, do vary, which was pointed out in both experiments [39–42] and empirical theories [6,10]. It is important to note here that confined plasma is a non-thermodynamic open system in which the plasma is maintained by an external source and sink. Hence, the induced heat flux *Q*_0_ is nearly constant in the source/sink-free region in quasi-steady state, which features in figure 1*b*. Considering that *Q*_0_ flows-in with higher temperature, $T_{h}$, near the core while heat flux flow-out occurs at lower temperature near the edge, an available entropy production rate is also constrained to the value which compensates the negative entropy production rate given by $(1/T_{h}-1/T_{c})Q_{0}$, [43,44]. Meanwhile, nonlinear flux-tube simulations using flux matching techniques have successfully reproduced the diffusivity distribution in L-mode plasmas [45–47], while there is intensive debate as to whether the essential properties of L-modes are of local or global origin in nature. It is important to know whether profile constraints observed in experiments originate from the characteristics in a flux-driven system or more local phenomena.

Here, we study the mechanism that forms a constrained profile as discussed above by using the electrostatic (ES) version of GKNET covering the core to edge plasma as an open system, which models those in figure 1*b*, under the adiabatic electron response [29,33,39–41,48]. In the simulation using a monotonically increasing safety factor profile with gentle density gradient, we observe a typical constrained profile of exponential function form but with spatially constant two piecewise different scale lengths, i.e. inside is gentle while outside is steeper, which are connected smoothly at a certain point of the minor radius. Such a two-scale nature becomes more prominent with increasing input power. To extract the characteristics of the turbulent transport, we introduced a real space-based statistical method, i.e. size-probability density function (PDF), which directly measures the size distribution of the heat flux eddies [29]. From the size-PDF obtained from the series of simulations, we found that the size-PDF exhibits a piecewise power law $P^{-\alpha }$ depending on the region of eddy size *S*, where each region corresponds to different transport processes. Based on the analysis, we identified non-local and non-diffusive processes which are hardly described by the flux-gradient Fick's law and then quasi-linear hypothesis, and classified them into three processes, 1. *fast time scale, radially localized avalanches*, 2. *radially extended instantaneous global bursts* and 3. *short spatial scale E* × *B shear layers coupled with pressure corrugation and their slow time scale evolution*. Here, process 2 is a notable event which constitutes a quasi-deterministic, non-power tail component in the size-PDF, which extracts larger turbulent free energy in the system through larger structure than that of linear, and plays a role to initiate an *E* × *B* shear layer pattern in process 3. Note that process 3 is considered to be the same as the *E* × *B* staircase discussed above and in [30–36]. It is also noted that processes 1 and 2 exhibit different spatial scales but provide similar radial correlation lengths, effectively from meso-scale to macro-scale. Based on the idea of hierarchical relation of the global profile, we concluded that processes 1 and 2 lead to the effect of global constraint on the profile. It is also found that process 3, i.e. *E* × *B* shear layers, plays a role not only to improve the confinement as local micro-scale transport barriers, but also to adjust the global profile self-consistently as seen in the present simulation as that consisting of two piecewise constrained profiles, especially in higher input power regimes. Here, the inner region gentle gradient profiles are subject to a strong constraint, while the outer gradients are steeper, indicating the partial weakening of the constraint, showing a tendency towards confinement improvement although the overall state is in L-mode. The underlying physical mechanisms are discussed focusing on processes 1, 2 and 3.

We present results in §2a and analyse the relaxation dynamics in §2b. In §2c, we study the spatio-temporal dynamics of turbulence based on the size-PDF of heat flux eddies and discuss the background of profile constraint. Finally, we give the concluding remarks in §3.

## 2. Transport characteristics in flux-driven system

**Figure 2.**
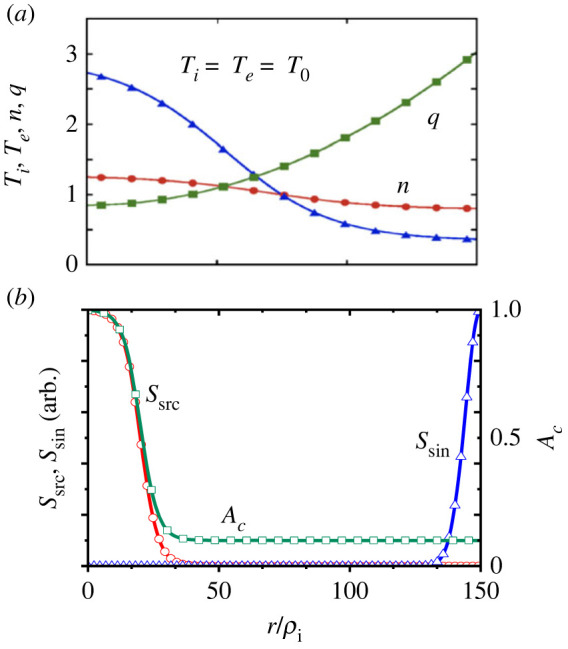
(*a*) Initial temperature and density profiles, $T_{i}(r)$ and $n_{i}(r)$, and safety factor profile q(r). (*b*) Profile of heating source and sink, i.e. $S_{\text{src}}$ and sink $S_{\text{sik}}$, and that of the form factor of the collisionality, $\nu_{\text{col}}(r)$. (Online version in colour.)

**Figure 3.**
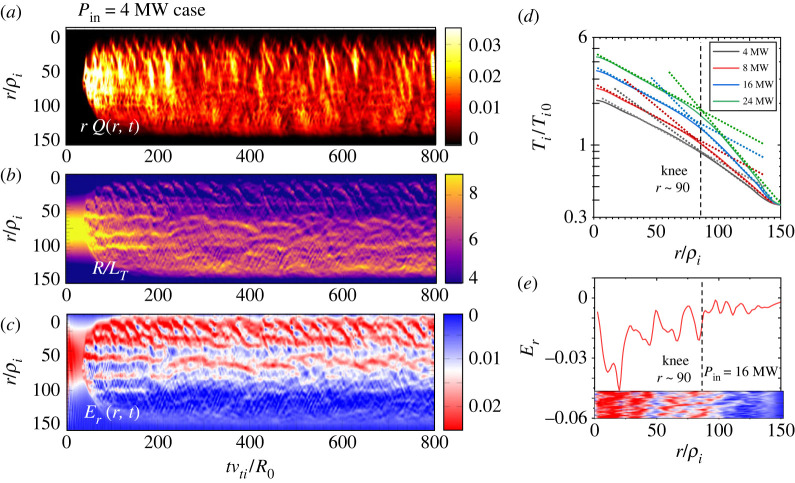
Evolution of distribution in (r,t) plane for (*a*) heat flux$\;rQ_{\text{turb}}$, (*b*) temperature scale length $R_{0}/L_{T}$, (*c*) radial electric field shear $E_{r}$ for $P_{\text{in}}=4\;\mathrm{MW}$ in the case with the form factor shown in figure 2*b*, i.e. $\nu_{*}=0.42$ in the core region.(*d*) Established temperature profiles in the quasi-stationary state for different input power of *P*_in_ = 4, 8, 16, 24 MW. Each profile has an exponential function fit, the scale lengths of which are changed at r∼90 defined by ‘knee’. (*e*) Distribution of $E_{r}$ for *P*_in_ = 16 MW, which corresponds to that in (*d*) and also figure 4, in which collisionality is also $\nu_{*}=0.42$ in the core region. In (*e*), the dynamics of $E_{r}$ is also inserted in 300≤t≤600 for reference. (Online version in colour.)

**Figure 4.**
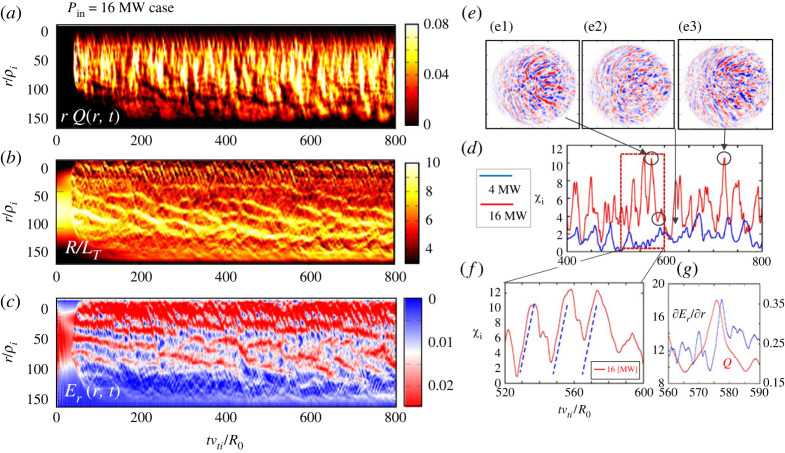
Evolution of distribution in (r,t) plane for (*a*) heat flux$\;rQ_{\text{turb}}$, (*b*) temperature scale length $R_{0}/L_{T}$ and (*c*) radial electric field $E_{r}$for $P_{in}=16\;\mathrm{MW}$ in the case with form factor shown in figure 2*b*, i.e. $\nu_{*}=0.42$ in the core region.(*d*) Corresponding radially averaged heat diffusivity $\chi_{i}$, (*f*) Heat diffusivity $\chi_{i}$ averaged in 520≤t≤600 and (*g*) heat flux *Q* as well as radial electric field shear $\partial E_{r}/\partial r$. (*e*) Represents potential contour plots on poloidal cross-section at times that the heat diffusivity $\chi_{i}$ becomes peak values for (*e*1) and (*e*3) while a bottom value for (*e*3). (Online version in colour.)

Based on the background discussed in §1, we organize the simulation results using the ES version of GKNET covering from core to edge as an open system, which models those in figure 1*b* [39–41,48]. The system represents a self-consistent global profile evolution sustained by an external heat source and sink. Here, we simulate ion temperature gradient (ITG) turbulence with adiabatic electron response in a circular plasma with $a/R_{0}=0.36$ and $a/\rho_{i0}=150$. Here, $\rho_{i0}$ is the gyro-radius for the ion thermal velocity $\upsilon_{i0}=\sqrt{{\left\langle T_{i}\right\rangle }_{0-a}/M}$, where ${\left\langle r\right\rangle }_{r_{1}-r_{2}}$ denotes the spatial average in the radial region $r_{1}\leq r\leq r_{2}$. The initial ion and electron temperature profile, i.e. $T_{i,e}=T_{0}(r)$, and safety factor profile given by $q(r)=0.85+2.18{\left(r\text{/}a\right)}^{2}$ are shown in figure 2*a*. Figure 2*b* shows the profile of source and sink terms, $S_{\text{src}}$ and $S_{\text{sik}}$. The normalized collisionality is chosen to $\nu_{*}=0.42$ in the source and sink-free regions using the factor *A_c_* as shown in figure 2*b*. In the following, time and space are normalized as $t\upsilon_{i0}/R\to t$ and $r/\rho_{i0}\to r$. Flux-driven simulations for the input powers of $P_{\text{in}}=4\;\text{MW}$ and $P_{\text{in}}=16\;\text{MW}$ are performed, for which results are shown in figures 3 and 4, respectively.

Figure 3 shows (*a*) turbulent heat flux $rQ_{\text{tub}}$, (*b*) inverse ion temperature scale length $R/L_{T}$ and (*c*) radial electric field $E_{r}$ in (r,t) space for $P_{\text{in}}=4\;\text{MW}$. In (*d*), we also show the logarithmic plots of ion temperature profile, $T_{i}$, averaged in the quasi-steady state for *P*_in_ = 4, 8, 16 and 24MW, respectively. Figure 3*e* shows the radial distribution for $P_{\text{in}}=16\;\text{MW}$, which corresponds to the 16 MW case in figure 3*d* and also figure 4. Figure 4 shows the corresponding quantities for $P_{\text{in}}=16\;\text{MW}$, i.e. (*a*) $rQ_{\text{tub}}$, (*b*) $R_{0}/L_{T}$ and (*c*) $E_{r}$. The time histories of the heat diffusivity $\chi_{i}$ averaged over the source and sink-free region are illustrated in figure 4*d* for both $P_{\text{in}}=4\;\text{MW}$ (blue) and 16 MW (red). Figure 4*f* shows the logarithmic plot of total ion heat diffusivity $\chi_{i}$ with the neo-classical component over the duration of three repetitive bursting phases. The relationship between the heat flux $Q_{\text{tub}}$ and the radial electric field shear $\partial E_{r}/\partial r$ in one burst phase is shown in figure 4*g*. We also show the contour plots of ES potential ϕ(r,θ) at times that the heat diffusivity $\chi_{i}$ peaks for (*e*1) and (*e*3), and a minimum value for (*e*2) (indicated by arrows). In the following, we describe the characteristics of transport based on figures 3 and 4.

### (a) Three types of meso-scale to macro-scale transport events

Figures 3 and 4 exhibit complex transport dynamics with a mixture of multiple elementary processes with different spatio-temporal scales. To characterize such dynamics, as we describe in §2c (also see [29]), we explored a real space (not Fourier space)-based statistical method to directly measure the size distribution of heat flux eddies (figure 13), which is expressed by the PDF *P*(*S*) with *S* the eddy size (area). In this method, we define each heat flux eddy designated by ‘*i*’ on the poloidal cross-section (i=1∼N), which contains relevant information such as eddy size *S_i_*, heat flux $\delta Q_{i}$ and heat flux density $D_{i}\text{/ }S_{i}$. Note that there are ± quantities such as $S_{i}^{\pm }$ and $\delta q_{i}^{\pm }$ corresponding to the outward and inward heat fluxes. As shown in §2c (figure 14), we found that the size-PDF exhibits a piecewise power law dependence as $P\propto S^{\alpha }$ with exponent α depending on the region of *S*. Based on the property of the quasi-linear theory that the residue of ± heat flux, i.e. $\sum_{\pm }\delta q_{i}^{\pm }$, gives rise to the net heat flux, one can see that the diffusive transport is induced by heat flux eddies with sizes in the range $1\leq S\leq S_{a}$ (region (*a*)). In the present system size of a=150, we evaluate as $S_{a}∼40$. This is because the size-PDF containing the region of S=1 is dominated by a single power law dependence with α=2/3 as $P\propto S^{-2/3}$. It is also confirmed that the quasi-linear trend is well satisfied in this region, as seen in figure 13*b*1, where the net heat flux in the region [1−S] monotonically (adiabatically) increases with *S* up to $S∼S_{a}∼40$. On the other hand, we found that the regions with larger eddy size for $S_{a}\leq S$ are regulated by different power laws, where adiabatic cancellation rule breaks down. Therefore, considering that the linear flux-gradient Fick's law and associate quasi-linear hypothesis can hardly be applied, we may define transport events governed by eddy sizes.

Based on the above idea, we classify observed non-local and non-diffusive events in figures 3 and 4 according to three fundamental processes with different spatio-temporal scales, i.e. 1. *the fast time scale radially localized avalanches*, 2. *radially extended instantaneous global bursts* and 3. *short spatial scale E × B shear layer formation coupled with pressure corrugation*. Here, process 3 exhibits a complex behaviour, especially in the higher power regimes, leading to long-time scale transport. It is noted that process 1 is considered to emerge in $S_{a}\leq S\leq S_{b}$ (region (*b*)) while process 2 is in $S\geq S_{b}$ (region (*c*)), where $S_{a}∼40$ and $S_{b}∼180$ are estimated. Meanwhile, regions (*b*) and (*c*) are found to be regulated by $P\propto S^{-2}$ and $P\propto S^{-4}$, respectively in the quiescent phase. However, the power law dependence is found to be violated during the bursting phases. In the following, we describe each process.

#### (i) Fast time scale radially localized avalanche

**Figure 5.**
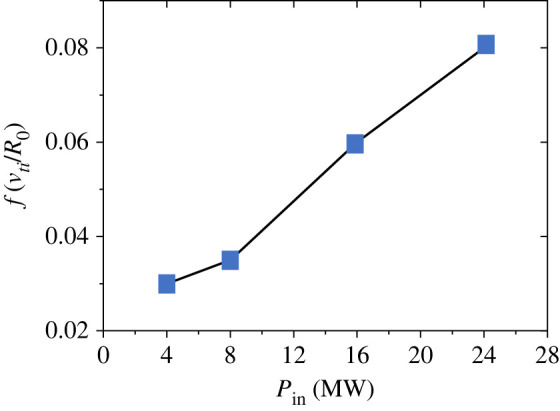
Frequency of fast time scale avalanches in the central heating region as a function input power. (Online version in colour.)

An avalanche is recognized as the propagation of short wavelength pressure perturbation, $\delta T_{i}$, which is widely observed in the flux-driven simulations [24–26,29,33]. Two types of avalanches, a hump which propagates downwards, while a hole propagates upward, can be seen depending on the sign of *E_r_* shear. The former results in $\partial_{r}E_{r}>0$, while the latter in $\partial_{r}E_{r}<0$, respectively [42]. The radial scale of avalanches shows a distribution, while those with $\Delta r_{\text{av}}=10∼20$ propagating with $\upsilon_{\text{fav}}∼3v_{d}$ are widely observed. Here, $v_{d}=(\rho_{i}\text{/}R)\upsilon_{i}$ is the magnetic drift velocity. As discussed above, region (*b*) is regulated by eddies with the size of 40≤S≤180, so that the corresponding size in the poloidal direction is estimated as $\Delta \ell_{\theta }=4∼8$, which is consistent to those observed in quiescent phase in figures 3 and 4. Assuming that the avalanche propagates over the temperature scale length, the corresponding time scale is $\Delta \tau_{\text{av}}∼0.3\;(L_{T}/\upsilon_{d})$. Meanwhile, the event is clearly observed in the heating region 0≤r<30 near the centre, as seen in figure 3*a* and figure 4*a*. The events are triggered quasi-periodically and supply turbulence energy to the bulk region. The frequency is found to increase with heating power as shown in figure 5.

#### (ii) Radially extended instantaneous global bursts

This is also a typical event observed in toroidal simulations. The radial size, $\Delta r_{\text{gb}}$, of the event is distributed from meso-scale to macro-scale, leading to $\Delta r_{\text{gb}}=20∼100$ seen in figure 3*a* and figure 4*a*. This corresponds to a range $\Delta r_{\text{gb}}={\left(\rho_{i}L_{T}\right)}^{1/2}∼L_{T}$. It is noted that the large scales of the order of *L_T_* exceed those of linear eigen-mode which is around ${\left(\rho_{i}L_{T}\right)}^{1/2}$. The typical lifetime of the burst is estimated as $\Delta \tau_{\text{bust}}∼10R/\upsilon_{i}$, which is rewritten as $\Delta \tau_{\text{bust}}∼0.1(L_{T}/\upsilon_{d})$ using $L_{T}/\rho_{i0}∼100$ (from figure 3*d*). This is found to be the same order as process 1, i.e. $\Delta \tau_{\text{av}}∼0.3$ $\left(L_{T}/\upsilon_{d}\right)$, indicating that the effect of avalanche and global bursts on the constraints for the profile formation and relaxation is expected to be similar, which is used in the discussion for the constraints in §2b.

Meanwhile, as seen in both figure 3*a* and figure 4*a* as well as figure 4*d,f*, the burst repeats with a period of $\tau_{\text{b - rep}}∼20R/\upsilon_{i}$, which is the same order as $\tau_{\text{bust}}$. This event is found to be due to the instantaneous phase alignment of smaller scale potential eddies in the radial direction and subsequent nearly exponential growth, leading to a radially extended structure with nearly up-down symmetry in the poloidal cross-section seen in figure 4*d,f,g*, as well as figure 4*e*1,*e*3, which show the ES potential distribution near the saturation, while figure 4*e*2 shows the state after the break-up.

The mechanism is studied qualitatively by the first-order ballooning theory with respect to 1/*n*, where *n* is the toroidal mode number, which takes into account the effect of the global profile variation as well as that of the equilibrium $E_{r}$ field [49,50]. Here, the radial envelope width and tilting (Bloch) angle of the toroidal ITG mode from the mid-plain, $\left(\Delta r,\theta_{0}\right)$, are given by

2.1 $$\Delta r∼{\left|\frac{2\gamma_{0}\sin\theta_{0}}{k_{\theta }\hat{s}\partial_{r}(\omega_{d}+\omega_{E})}\right|}^{1/2}$$

and

**Figure 6.**
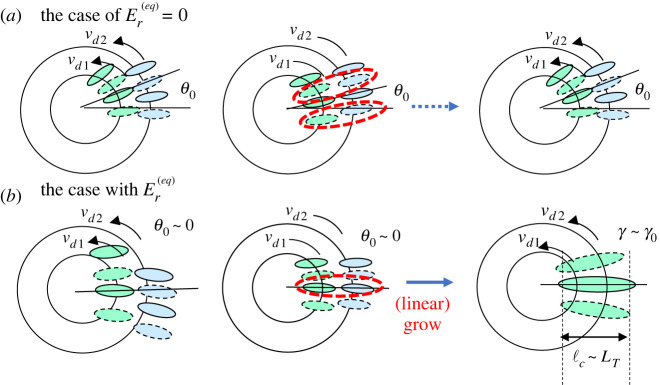
Schematic view of instantaneous phase matching in the absence of equilibrium $E_{r}$ field (*a*) with finite tilting angle $\theta_{0}>0$ due to the effect of global profile variation, while the structure fails to grow. Case (*b*) is the case with equilibrium $E_{r}$ field, leading to $\theta_{0}∼0$, which trigger the growth of structure. (Online version in colour.)

2.2 $$\theta_{0}=\pm {\left|\frac{\partial_{r}(\omega_{d}+\omega_{E})}{2k_{\theta }\gamma_{0}\hat{s}}\right|}^{1/3},$$

respectively, where $\omega_{E}=k_{\theta }v_{E}$ with $v_{E}=E_{r}^{\left(\text{eq}\right)}/B$, $\omega_{d}=(k_{\theta }\rho_{i})v_{i}/R_{0}=k_{\theta }v_{d}$ and $\gamma_{0}$ the growth rate obtained from the zeroth-order ballooning theory [51]. Here, $\theta_{0}$ represents the up-down asymmetry of the toroidal ITG mode on the poloidal cross-section, by which the growth rate is given by $\gamma ∼\gamma_{0}\cos\theta_{0}$ [49,50]. It is also known that $\theta_{0}$ is related to the residual toroidal momentum flux [52]. The tilting of the mode is induced by the diamagnetic drift frequency shear $\partial_{r}\omega_{d}$ due to the global profile variation. However, it can be partially cancelled by the presence of the equilibrium $E_{r}^{\left(\text{eq}\right)}$ field and lead to $\partial_{r}(\omega_{d}+\omega_{E})∼0$ when the cancellation is complete and then $\theta_{0}∼0$. In this case, the up-down symmetrical structure is found to be recovered as seen in figure 4*e*1,*e*3. This event not only maximizes the growth rate to $\gamma ∼\gamma_{0}$, but also increases the radial mode width as found from Equation (2.2), i.e. Δr→∞. This corresponds to the generation of large size eddies in the region 180≤S as seen in figure 14*a*2,*b*2, i.e. bursting phase, in which the maximum eddy size reaches S∼1000. This feature is schematically illustrated in figure 6 for both cases with and without equilibrium $E_{r}^{\left(\text{eq}\right)}$ field. The process is found to be repeated and induces an *E* × *B* shear layer as discussed in process 3.

The above discussion is based on the linear aspect, then the application to the dynamics in the quasi-steady nonlinear phase is not straightforward. However, the avalanche dynamics is considered to be those in near marginal state [24,53] where the linear process and that of nonlinear are competing. Therefore, it is worth applying, as one of analysis methods assumes the situation that linear modes grow in a fluctuating field.

#### (iii) Formation of *E* × *B* shear layer and dynamics

This is a meso-scale quasi-regular *E* × *B* shear layer coupled with pressure corrugation [33,35] as typically seen in figure 3*b,c* for $P_{\text{in}}=4\;\text{MW}$. The spatial interval of the corrugation is estimated as $\Delta r_{sc}∼20\rho_{i}$, as seen in figure 3*e*, which is thought to be essentially similar to those referred to as an *E* × *B* staircase [30,31]. The formation mechanism as well as the effect on the transport have been intensively discussed. The pattern is quasi-regular and relatively stable especially near the heating boundary at *r* = 30–50, which shows unstable behaviours with breaking, splitting and merging from the core to edge region. Meanwhile, in the higher power case $(P_{\text{in}}=16\;\text{MW) }$, the structure is found to propagate outward with an average velocity of around $\upsilon_{\text{s - av}}=0.2-0.3v_{d}$, an order of magnitude slower than process 1, the fast time scale avalanches, as seen in figure 4*b,c*. The E × B staircase is therefore regarded as a slow time scale avalanche. In the following, we study the mechanism of these processes.

**Figure 7.**
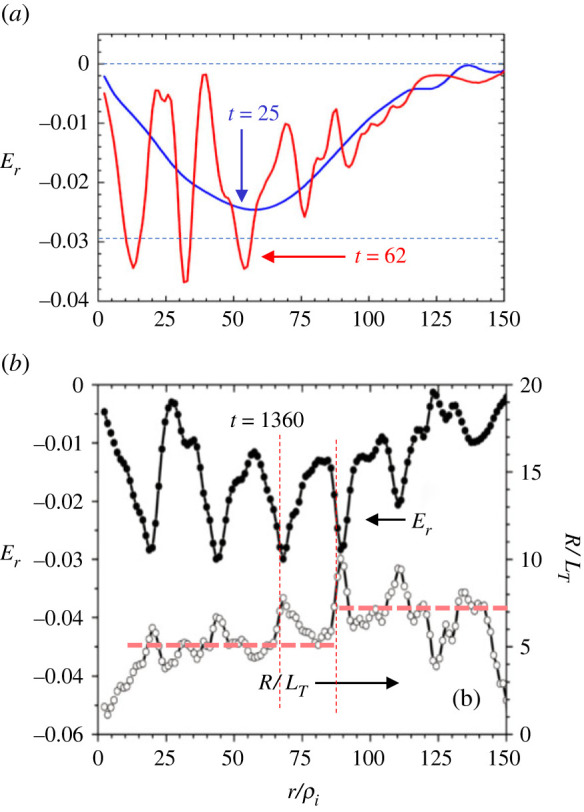
(*a*) Distribution of radial electric field $E_{r}$ before linear ITG mode grows at *t* = 25 and after the mode saturate at *t* = 62, (*b*) distribution of $E_{r}(<0)$ and $R/L_{T}$ in the quasi-steady state at *t* = 1360 (data from [33]). (Online version in colour.)

**Figure 8.**
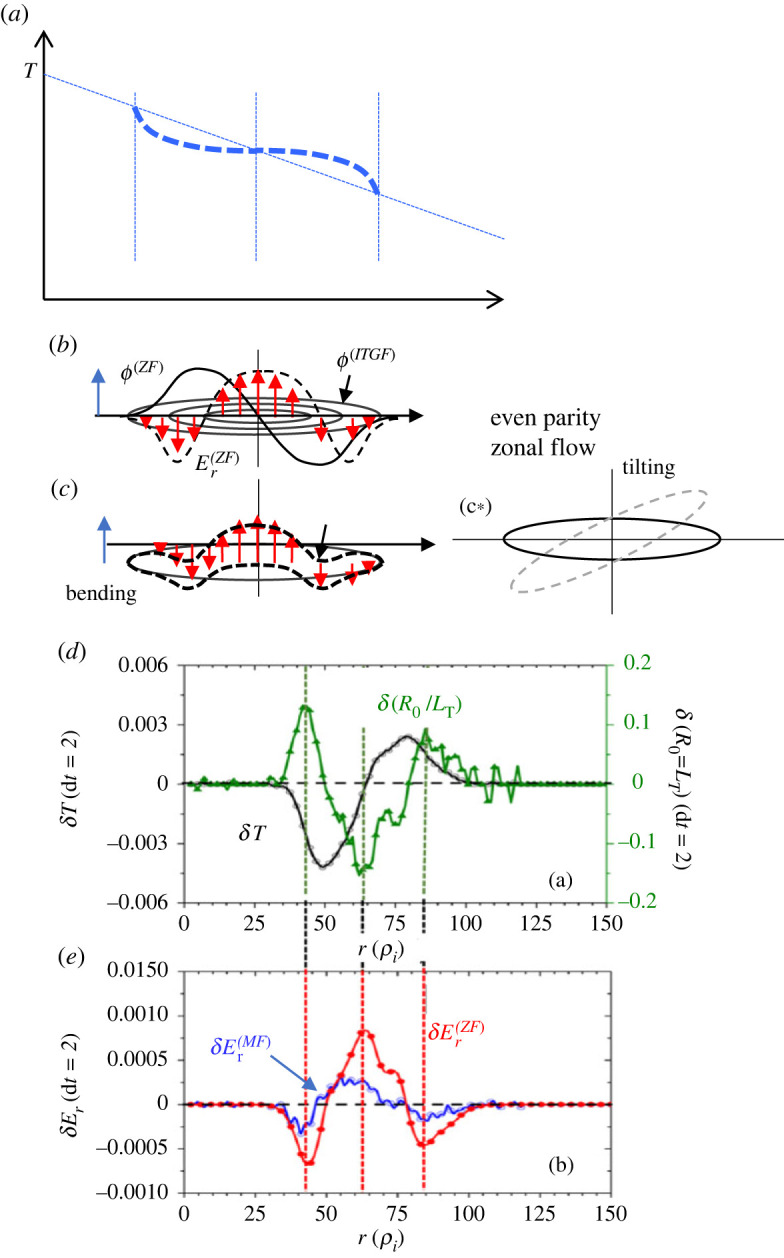
Schematic picture of (*a*) temperature relaxation due to the meso-scale ITG mode, (*b*) meso-scale ITG mode structure and (*c*) distortion by self-generated zonal flow with even parity. Suppression and saturation take place due to not tilting like (c_*_) but by bending. (*d*) Temperature relaxation δT (difference from the initial profile) and corresponding that of $\delta (R_{0}/L_{T})$ due to the ITG mode near saturation of ITG mode from the δf simulation of GKNET. (*e*) The produced radial electric field due to the zonal flow and temperature relaxation given in (*d*), which show in-phase relation (data: those from [33]). (Online version in colour.)

As seen in figure 7*a*, a global equilibrium field $E_{r}^{\left(\text{eq}\right)}$ is formed on the fast time scale at *t* = 25 before the ITG mode growth. Subsequently, the toroidal ITG modes grow and saturate at around *t* = 62, while the radial electric field *E_r_* shows an oscillatory distribution in the entire region, indicating the generation of the $\delta E_{r}$ field which is superimposed to the equilibrium field $E_{r}^{\left(\text{eq}\right)}$. The $\delta E_{r}$ field is considered to consist of two parts, one is that due to the induced zonal flow and the other is that due to the temperature relaxation $\delta T_{i}$, which we formally represent as $\delta E_{r}^{\left(ZF\right)}$ and $\delta E_{r}^{\left(TR\right)}$, respectively. As explained later in this section, $\delta E_{r}^{\left(ZF\right)}$ represents the equilibrium part affected by the zonal flow damping. Since these two events take place almost simultaneously, they are not strictly separated. However, it is worth considering the relative relation, the feature of which is outlined schematically in figure 8*a–c*. The result obtained from the δf version of GKNET is also shown in figure 8*d*. Here, it is noted that the zonal flow part $E_{r}^{\left(ZF\right)}$ and associated *E* × *B* flow $v_{E}^{\left(ZF\right)}=\delta E_{r}^{\left(ZF\right)}/B$ exhibit an *even parity* function as shown in figure 8*b*. This is evaluated from the Hasegawa-Mima equation as that shown in figure 8*c* (see equations (11)–(13) in [33]). Namely, it is found that the direction of the driven *E* × *B* flow is reversed between the central region of the ITG mode and both edges, which exhibits an even function. Therefore, the associated deformation of the ITG mode is not tilted but curved, as shown in figure 8*c*. Note that the corresponding zonal potential $\phi^{\left(ZF\right)}$ exhibits an odd parity function.

On the other hand, the part due to the pressure relaxation, i.e. $\delta E_{r}^{\left(TR\right)}$, estimated by applying the change of the temperature profile due to the relaxation before $\left(t=t_{1}\right)$ and after $\left(t=t_{2}\right)$ to the radial force balance relation as follows [33]:

2.3 $$\delta E_{r}^{\left(TR\right)}\approx {\left[-\frac{T_{i}}{e}\left(\frac{1}{L_{n}}+\frac{1-k}{L_{T}}\right)+\frac{rB}{qR}U_{∥}\right]|}_{t_{1}}^{t_{2}},$$

where both ion density and temperature are assumed to be exponential functions with the scale length of $L_{n}$ and $L_{T}$. The result is shown in figure 8*e*, where $\delta E_{r}^{\left(TR\right)}$ exhibits not only an even parity function but also in-phase relation with the zonal electric field $\delta E_{r}^{\left(ZF\right)}$, indicating that the zonal flow part and that due to temperature relaxation reinforce each other. We represent the total perturbation field by these two processes as $\delta E_{r}^{\left(ZF\right)}+\delta E_{r}^{\left(TR\right)}\to \delta E_{r}^{\left(ITG\right)}$. Note that the effect of temperature relaxation is approximately 1/3 of that due to zonal flows. The total field is given by adding the equilibrium part as $E_{r}^{\left(\text{tot}\right)}∼E_{r}^{\left(\text{eq}\right)}+\delta E_{r}^{\left(\text{ITG}\right)}$.

In the quasi-steady state at *t* = 1360, similar oscillatory distribution to that at *t* = 62 is found to be maintained, but developed due to a spiky structure at each peak of the field. It is also found that $E_{r}^{\left(\text{tot}\right)}$ couples with pressure corrugation as seen in the distribution of $R/L_{T}$ in figure 7*b*, and $E_{r}^{\left(\text{tot}\right)}$ and $R/L_{T}$ exhibit a clear (face to face) in-phase relation. Namely, the steepening of the temperature profile is regulated not by a simple turbulent suppression due to the $E_{r}^{\left(\text{tot}\right)}$ shear, but by the balance with the $E_{r}^{\left(\text{tot}\right)}$ field itself through radial force balance relation. This is also consistent with the fact that $\delta E_{r}^{\left(ZF\right)}$ and $\delta E_{r}^{\left(TR\right)}$ are in-phase, and $\delta E_{r}^{\left(TR\right)}$ is determined so as to satisfy the radial force balance relation. It is also worth noting the role of zonal flow damping. That is, the zonal flow and associated radial electric field are affected by either collision-less damping or collisional effects. However, in the present case, induced zonal electric fields are partly assimilated into the equilibrium field $\delta E_{r}^{\left(ZF\right)}$ coupled with the temperature relaxation $\delta T_{i}$, so that $\delta E_{r}^{\left(ZF\right)}$ satisfies the radial force balance equation before undergoing zonal flow damping. Here, it is noted that the temperature perturbation, $\delta T_{i}$, is the dominant counterpart in the balance relation, while toroidal and (neo-classical) poloidal rotations are also induced.

**Figure 9.**
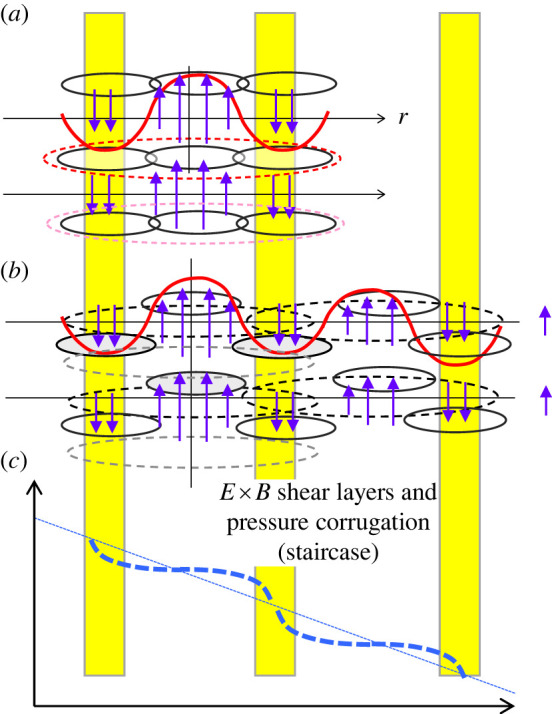
(*a*) Feature of the break-up of meso-scale potential eddy due to self-generated $E_{r}$ field with even parity and reconstruction of meso-scale eddy due to the subsequent phase matching. (*b*) Connection of different eddies in radial direction leading to multiple staircase as shown in (*d*). (data from [33]). (Online version in colour.)

Here, the in-phase relation between $\delta E_{r}^{\left(ZF\right)}$ and $\delta E_{r}^{\left(TR\right)}$, which are both generated from the radially extended ITG mode, is the key to sustain the structure as seen in figure 8. The bending of radially extended ITG mode due to the $E_{r}^{\left(\text{tot}\right)}$ eventually disintegrates (splits) the extended structure into three pieces as seen in figure 9, which saturates the growth of the ITG mode leading to the damping. Meanwhile, such disintegrated potential eddies rotating in different poloidal directions are aligned after a time interval approximately estimated by $\tau_{b-\text{rep}}∼2\pi \text{/}k_{\theta }v_{E}$ due to the phase alignment in the radial direction, where $v_{E}∼E_{r}^{\left(\text{tot}\right)}/B$ represents the total *E* × *B* flow velocity. This structure can be an initial condition from which radially extended ITG modes with nearly up-down symmetry with $\theta_{0}∼0$ grow, as discussed in process 2, and in figure 9 (also figure 6). A series of processes, including (a) the phase alignment of potential eddies located at different radii and subsequent exponential growth to larger amplitude, (b) generation of the $E_{r}$ field due to an in-phase relation between zonal flow and pressure relaxation, $E_{r}^{\left(ZF\right)}$ and $E_{r}^{\left(TR\right)}$ and (c) disintegration of radially extended eddy leading to mode saturation and damping, is repeated, which causes quasi-period bursts. The repetition of such bursts sustains the peaking structure of *E* × *B* shear layers self-consistently against the damping of $E_{r}$, especially for perturbed parts, i.e. $E_{r}^{\left(ITG\right)}=E_{r}^{\left(ZF\right)}+E_{r}^{\left(TR\right)}$, as observed in figures 3 and 4. This can be seen as the radially extended ITG modes being trapped and/or sandwiched between *E* × *B* shear layers. Such a structure is also coupled in the radial direction leading to multiple *E* × *B* shear layers as schematically shown in figure 9*d* and also those seen in the simulation in figures 3 and 4. Such *E* × *B* shear layers are not stable even in the lower power regime of $P_{\text{in}}=4\text{MW}$, showing transient dynamics such as breaking, splitting and merging are observed. Furthermore, they evolve and/or propagate in the radial direction towards the edge on average in the higher power regime of $P_{\text{in}}=16\text{MW},$ as seen in figure 4, which repeats at time intervals of $\Delta t=100-200R/\upsilon_{i}$, leading to a long-time scale breaking in transport.

**Figure 10.**
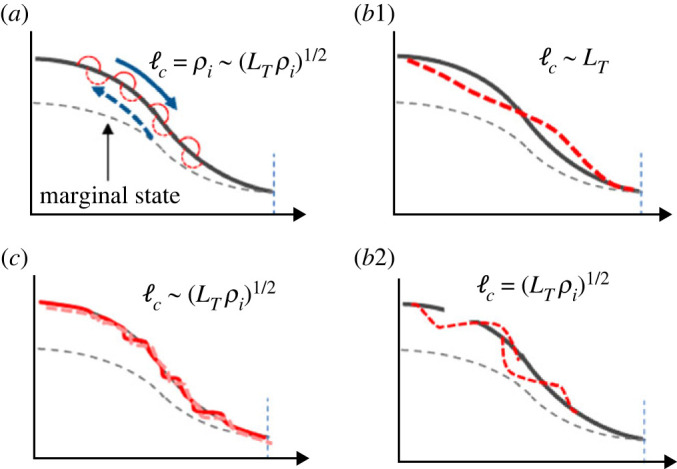
Schematic picture showing three types of non-non-diffusive transport process, (*a*) fast time scale avalanche, (*b*1) and (*b*2) two type of radially extended global bursts, (*b*1) global burst over the scale length and (*b*2) those of meso-scale and (*c*) E×B shear layers coupled with pressure corrugations (staircase). Typical scales of each event are given. (Online version in colour.)

These three types of transport event are schematically illustrated in figure 10, where (*a*) corresponds to process 1, fast time scale spatially localized avalanche, and (*b*1) (*b*2) to process 2, global bursts with different two scales, i.e. (*b*1) global scale over the temperature scale length while (*b*2) those of meso-scale, (*c*) to process 3, short scale *E* × *B* shear layers, respectively.

### (b) Constraint for profile formation and relaxation

**Figure 11.**
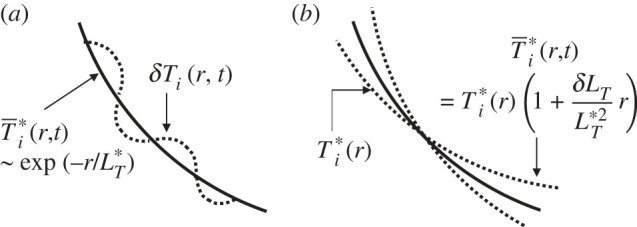
Schematic picture of hierarchized relaxation process. (*a*) The global constrained self-organized profile $T_{0}$ and smaller scale perturbation $T_{1}$ on it and (*b*) global relaxation of the self-organized profile $T_{0}$.

As described in §2a, due to the strong constraint that the temperature scale length becomes spatially constant, an initially set arbitrary temperature profile *T_i_*(*r*) ( figure 2*a*) is relaxed to the exponential function form given by,

2.4 $$T_{i}^{*}(r)∼\exp\left(-\frac{r}{L_{T}^{*}}\right),$$

where $L_{T}^{*}$ is spatially constant over longer spatial scales on the order of temperature scale length $r∼r_{1}$ [51]. This feature is observed at different input powers *P*_in_ from 4 MW to 24 MW in the quasi-steady state, as shown in figure 3*d*. Here, the formed profiles exhibit an interesting feature. For instance, for $P_{\text{in}}=16\text{MW}$, the profile is characterized by two different scale lengths, i.e. it is longer in the inner region $L_{T}^{*\text{in}}$ while shorter in the outer region $L_{T}^{*\text{out}}$, which change at $r∼r_{*}∼90$. We refer to it as *knee* point (region). As *P*_in_ increases, while maintaining the exponential function form both inside and outside the knee point, the inner scale length $L_{T}^{*\text{in}}$ remains unchanged, while the outer scale length $L_{T}^{*\text{out}}$ shortens. As a result, the core temperature $T_{i}(0)$ rises as seen in figure 3*d*. This indicates that the confinement becomes higher in the outer plasma compared with the inner region. In the following, we study the relaxation dynamics focusing on one of two piecewise regions. The corresponding dynamics are schematically shown in figure 11.

After the formation of the exponential function form as $L_{T}(r,t)\to L_{T}^{*}$, which is spatially constant, the same amount of heat flux, e.g. 4 MW, has to be sustained in the system while maintaining the same exponential function form on average. Assuming that the heat flux is sustained by non-local and/or non-diffusive processes with temperature perturbation, we express the instantaneous temperature profile as

2.5 $$T_{i}={\bar{T}}_{i}^{*}(r,t)+\delta T_{i}(r,t),$$

where $\delta T_{i}(r,t)$ is the temperature perturbation with various spatio-temporal scales. On the other hand, ${\bar{T}}_{i}^{*}(r,t)$ is responsible for the constrained profile corresponding to equation (2.4), but includes a global perturbation through the variation of temperature scale length as ${\bar{L}}_{T}^{*}\equiv \;L_{T}^{*}+\delta L_{T}(t)$, where $L_{T}^{*}$ is spatio-temporally constant, while $\delta L_{T}(t)$ is spatially constant but temporally variable. Then, it is expressed as

2.6 $${\bar{T}}_{i}^{*}(r,t)=T_{i}^{*}(r)\left(1+\frac{\delta L_{T}}{L_{T}^{*2}}r\right).$$

As discussed in §2a, process 1, i.e. the fast time scale avalanche, satisfies $\Delta r_{\text{av}}/L_{T}\ll 1$, whereas process 2, i.e. the radially extended global burst, satisfies $\Delta r_{\text{gb}}/L_{T}∼1$. Therefore, it is likely that process 1 is responsible for $\delta T_{i}(r,t)$ and process 2 for $\delta L_{T}(t)$ over the temperature scale length. However, $\delta T_{i}(r,t)$ is considered to have a broad wave number spectrum including those with long wavelengths. On the other hand, as discussed in §2a, both processes 1 and 2 have a characteristic to influence the long wavelength perturbation on a faster time scale on the order of $\Delta t∼\beta (L_{T}/\upsilon_{d})$ with β=0.1−0.3, which is on a similar time scale as that of drift wave. Here, β is estimated from $\Delta \tau_{\text{bust}}$ and $\Delta \tau_{\text{av}}$. Therefore, process 1 also regulates the dynamics for $\delta L_{T}$. This suggest that the global constraint on the profile is still effective even in the absence of process 2 such as global bursts.

**Figure 12.**
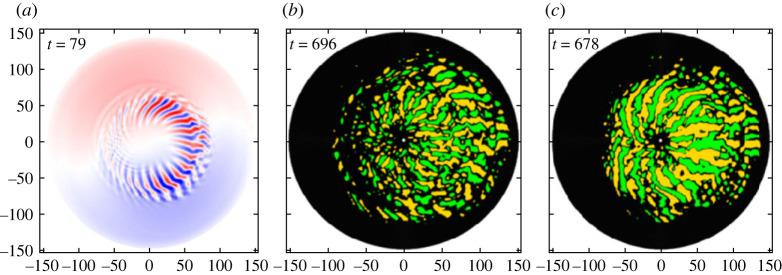
(*a*) Potential distribution of liner ITG mode at *t* = 79 corresponding to figure 13, (*b,c*) are distributions of heat flux eddies at *t* = 696 (quiescent phase) and *t* = 678 (bursting phase) corresponding to figure 14, respectively. (Online version in colour.)

### (c) Statistical study using size-probability density function of heat flux eddies

Here, we further study the turbulent transport discussed in §2a and 2b by using the size-PDF for the heat flux eddies, which is also explained in §2a. Based on the background of the method and unique characteristics of power law dependence, we describe the details in the following: figure 12 shows (*a*) potential distribution of liner ITG mode at *t* = 79 for $P_{\text{in}}=16\;\text{MW}$ presented in figure 4, *b* and *c* are distributions of heat flux eddies at *t* = 696 corresponding to figures 14*a*1,*b*1 (quiescent phase) and those at *t* = 678 to figures 14*a*2,*b*2 (bursting phase), respectively.

**Figure 13.**
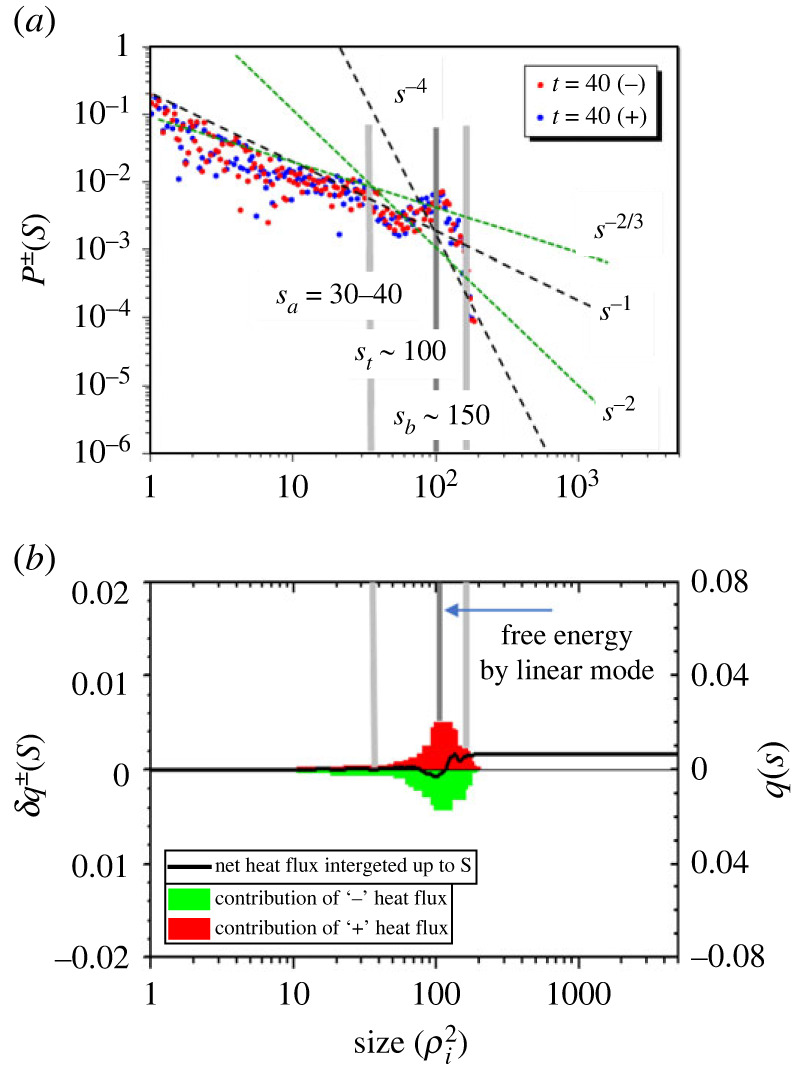
(*a*) Size-PDF for heat flux eddies $P^{\pm }(S)$ and (*b*) corresponding heat flux $\delta q^{\pm }(S)$ as a function of the heat eddy size *S* for typical ITG mode in linear phase at t=40 for $P_{\mathrm{in}}=16\;\mathrm{MW}$ (figure 12*a*). Red and green colours represent the positive and negative value. The black line in (*b*) represents the total heat flux in q(S) in [0∼S]. (Online version in colour.)

Figure 13*a* shows the distribution of the size-PDF $P_{i}^{\pm }(S_{i}^{\pm })$ for the linear stage of the ITG mode. Here, the area $S_{i}^{\pm }$ is normalized by $\rho_{i}^{2}$ and the signs ± denote positive (red: outward) and negative (green: inward) heat flux, indicating that each eddy designated by ‘*i*’ involves phase information between potential fluctuation and that of temperature. Figure 13*b* shows the corresponding heat flux distribution defined by $\delta q^{\pm }(S_{i}^{\pm })=\underset{S∼S+dS}{\sum }q_{i}^{\pm }$, where $q_{i}^{\pm }$ represents the induced heat flux by the eddy with the size $S_{i}^{\pm }$. The net heat flux summed up in the range [0∼*S*] is given $q(S)=\sum_{\pm }q^{\pm }(S)$ with $q^{\pm }(S)=\int_{0}^{S}\delta q^{\pm }(S)\text{d}S$, which is shown by the solid black line in figure 13*b*1. Note that the total heat flux is given by $q_{\text{tot}}=q(S\to S_{\text{max}})$, where $S_{\text{max}}$ is the maximum eddy size in the system. It can be seen that $q^{+}(S)$ and $q^{-}(S)$ are cancelled predominantly while the residue provides a net heat flux q(S) in [0,*S*]. Not only the heat flux, but also the heat flux density given by $D_{i}^{\pm }\equiv q_{i}^{\pm }/S_{i}^{\pm }$ can be estimated, which is a useful method to study the validity of quasi-linear hypothesis [29]. In figure 13*a*1 and *b*1, a hump in $P^{\pm }(S)$ and heat flux in $\delta q^{\pm }(S)$ can be seen in the region 60≤S≤180 with a peak at *S* ∼ 100, which correspond to linear ITG mode. Assuming that the eddy size is given by $S∼\ell_{s}\lambda_{\theta }/2=\pi \ell_{s}/k_{\theta }$, the typical radial mode width is estimated as $\ell_{s}∼k_{\theta }S/\pi$, which is used as a major of radial correlation length. Here, $\ell_{s}∼12.7$ is obtained for the eddy of *S* ∼ 100 using $k_{\theta }\rho_{i}=0.4$, which is consistent with that estimated from $\sqrt{\rho_{i}L_{T}}∼9$ with $L_{T}∼80$ $\left(R\text{/}L_{T}∼5\right)$. Here, *q*(*S*) increases monotonically, indicating that the quasi-linear hypothesis is approximately fulfilled.

**Figure 14.**
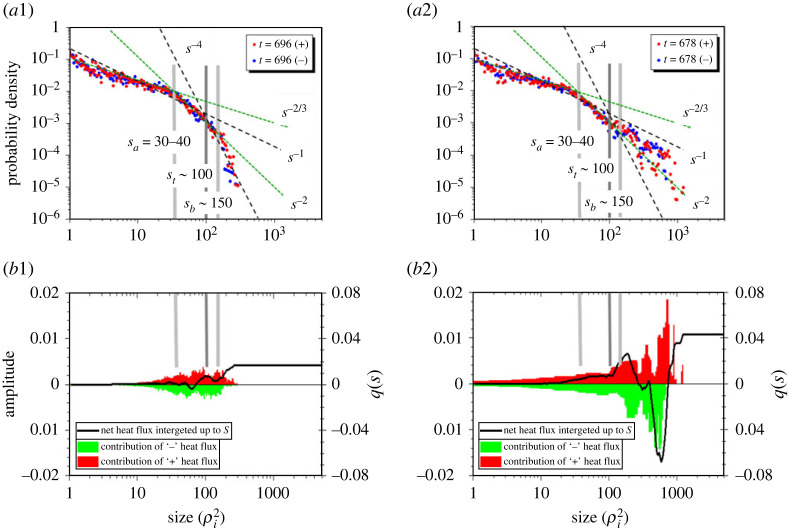
Size-PDF $P^{\pm }(S)$ and heat flux $\delta q^{\pm }(S)$ as a function of heat eddy size *S* at quiescent phase (t=696) in (*a*1) and (*b*1) corresponding to figure 12*b* and bursting phase (t=678) in (*a*2) and (*b*2) corresponding to figure 12*c*. (Online version in colour.)

Now, we study in a quiescent phase at *t* = 696 shown in figure 14*a*1,*b*1 and in a bursting phase at *t* = 678 in figure 14*a*2, *b*2, respectively. In the quiescent phase, the maximum eddy size is extended to $S_{\text{max}}∼300$, which is considered to be the inverse cascade. The forward cascade component is also seen in 20≤S≤60. The radial correlation length for *S*_max_ is estimated as $\ell_{s}∼38$, which is in the range from meso-scale to macro-scale. Meanwhile, the size-PDS, $P^{\pm }(S)$, is found to fit a three-piecewise power law as $P(S)∼S^{-\alpha }$, with two knee points at $S_{a}$ and $S_{b}$, which are given by the expression (*a*) $P∼S^{-2/3}$ for S≤30, (*b*) $P∼S^{-2}$ for 40≤S≤180 and (*c*) $P∼S^{-4}$ for 180≤S≤300, respectively. Here, the knee points are estimated as $S_{a}∼40$ and $S_{b}∼180$, with S∼100(5×20) the linear ITG mode (figure 13*a*1) in between. The radial correlation lengths for *S*_a_ and *S*_b_ are estimated as $\ell_{s(a)}∼5$ and $\ell_{s(b)}∼20$, respectively. The heat flux $\delta q^{\pm }(S)$ is also found to cancel up to S∼150, which is approximately the knee point *S*_b_, while it increases in S>150, as discussed above, which is in region (*c*), leading to the total net heat flux $q(S_{\text{max}})∼0.02$. This indicates that the heat flux is mainly sustained by a small number of larger size eddies in region (*c*) which exhibits $P∼S^{-4}$. This result is in contrast with the quasi-linear theory, which expects q(S) to increase monotonically with *S*.

Here, it is found that the quasi-linear relationship holds from smaller size of *S* = 1 up to $S∼S_{a}(=40)$, i.e. region (*a*), where the size-PDF exhibits a single power law $P∼S^{-2/3}$ containing the region *S* ∼ 1 where quasi-linear trend is justified. This is why region (*a*) belongs to that dominated by diffusive process in the discussion in §2a. In the bursting phase at t=678 in figure 12*c*, the size-PDF *P* as well as the net heat flux *q* is almost the same as the quiescent phase, and gives a similar power law, i.e. $P∼S^{-2/3}$ in region (*a*) with $S\leq S_{a}$ and $P∼S^{-2}$ in region (*b*) with $S_{a}\leq S\leq S_{b}$, respectively. However, the number of eddies increases significantly in the region (c*) with $S_{b}\leq S\leq S_{\text{max}}$, showing several non-power law irregular humps at $S∼200(\ell_{s}∼25)$, $400(\ell_{s}∼51)$, etc. If one fits the envelope peaks, it is located between $P∼S^{-2}$ and $P∼S^{-1}$, which greatly exceeds the quiescent phase in region (*c*) with $S^{-4}$ (figure 14*a*2). The maximum eddy size also increases to $S_{\text{max}}∼1000$, which means the radial correlation length is macro-scale, estimated as $\ell_{s}∼127$. This is comparable in size to the temperature scale length. This feature captures the characteristic of radially extended global burst discussed in process 2 in §2a. The net heat flux *q*(*S*) exhibits an oscillatory behaviour with respect to *S*, with nearly zero net flux at *S* ∼ 700, due to the cancellation. We found that a small number of larger size eddies present in 700≤S≤1000 is responsible for the total net heat flux around q∼0.04, i.e. twice the quiescent phase case. These features are far from quasi-linear trend, but exhibit rather quasi-deterministic and/or quasi-coherent aspects, which simply shows the dependence of $|q|\propto S^{2}$ and/or |D|∝S.

## 3. Discussion and concluding remarks

We studied dynamics leading to constrained (stiffness) profile and the underlying mechanism using the ES version of global toroidal gyro-kinetic GKNET simulations in a flux-driven open system, with source and sink in the framework of adiabatic electron response [29,33,39–41,48]. Some important processes such as those associated with trapped electron (TEM) and electromagnetic mode, e.g. kinetic ballooning mode (KBM) [53–55] are not taken into account, some of which are believed to be important in explaining transport shortfall events. However, it is rather meaningful to capture the properties of profile stiffness under conditions comparable to experiments based on minimum models as open systems. We observed two constrained regions of exponential profiles which are spatially constant over two piecewise different scale lengths. Here, the inner gradient is gentle while the outer region is steeper, and the two gradients are connected at about 2/3 of the minor radius. Such a two-scale nature of constrained profile shows a resemblance with those obtained experimentally [5–8,10,11]. However, the near-edge region is found to be sensitive to heating power and plasma current. A profile fit with a linear function that falls off faster than the exponential function is reported near the edge in experiments, which depends on the edge temperature [10].

In the present simulation, we regarded the region outside the knee region at r/a=1/2−2/3 as also subject to a constraint since it exhibits an exponential function in which scale length is spatially constant, except for the narrow sink region (0.9≤r/a≤1) closest to the edge ( figure 1). It is noted that the level of constraint is weak compared with those in the inner region where the scale length is almost unchanged with no dependence on the input power. This two-scale nature is also seen for the larger system size of $a=225\rho_{i0}$ where the scale separation is more valid [29]. As far as the mechanism, process 3, i.e. the slow time scale evolution of *E* × *B* shear layers towards the outside region as seen in figure 4*c*, can be considered. Namely, the $E_{r}$ field originates from the density perturbation. The downward convection of *E* × *B* shear layers accompanied by the pressure corrugation may play a role in accumulating the density perturbation in the outer region, which becomes again the origin of zonal flows. In fact, the increase of the $E_{r}$ shear in the outer region is observed in higher input power regime, which reduces transport and then shortens the global scale length. Another possible effect is the edge boundary condition. Here, we used a Dirichlet type boundary condition to model the outflow of the heat flux from the boundary. This is likely, while we introduce a sink term using a Krook model near the edge as shown in figure 2, which causes a distortion in distribution function. Once such effects propagate through the inner region, there could be a case in which the neo-classical transport increases. However, we have confirmed that the two-scale nature is weakly influenced by collisionality.

Meanwhile, one of the key results of this study is that it demonstrated the global profile formation and relaxation subject to a constraint leading to a basic feature of L-modes based on a minimum model as an open system, which are sustained only from the external action through source and sink. Therefore, the origin of the constraint is the central concern. As can be seen in figures 3 and 4, we observed complex dynamics with spatio-temporally different scales, which are classified as events not simply described by simple diffusive processes into three processes, i.e. 1. *radially localized fast time scale avalanche*, 2. *radially extended global burst*, 3. *meso-scale E × B shear layers and their slow time scale evolution*. To identify each event qualitatively or even quantitively, we explored a real space (not Fourier space)-based statistical method with size-PD, *P*(*S*), which represents the turbulent state as the size distribution of heat flux eddies in the system. We found that the size-PDF reveals a region-dependent piecewise power law given by $P^{-\beta }$ and each region is regulated by a particular power exponent to correspond to a physical process. Here, it is noted that the validity of the quasi-linear process can be studied from the behaviour of the PDF estimated heat flux distribution. Based on this study, we found that the above three processes are hardly described by the quasi-linear hypothesis and flux-gradient Fick's law and then considered as non-local and non-diffusive processes, especially for process 2. Namely, process 2 is found to be the result of the instantaneous phase alignment of small and/or meso-scale potential eddies in the radial direction and subsequent growth, leading to a radially extended ballooning-like structure with up-down nearly symmetry and then a burst. The structure is quickly disintegrated, while this process is repeated for different eddies located in the radial direction to be aligned in the next opportunity, the time scale of which is roughly estimated as $20(R_{0}/v_{T_{i}})$. Then, this process is repeated quasi-periodically, leading to intermittent global bursts. This process is manifested in size-PDF as a non-power law tail component. This is likely since the process is not fully probabilistic but includes a quasi-deterministic ingredient. This process is closely related to the formation of meso-scale *E* × *B* shear layers coupled with temperature corrugation with maintaining a (face to face) in-phase relation, i.e. the process 3, which is equivalent to an *E* × *B* staircase. The physical mechanism was discussed, which originates from the radial electric field *E_r_* and the shear which causes the saturation and subsequent break-up of the radially extended global bursts. Namely, it is noted that the *E* field consists of two ingredients, i.e. one that is due to zonal flows and the other that is due to global temperature relaxation through the radial force balance relation. Here, importantly, these two *E_r_* fields exhibit in-phase relation, so that they are maintained as *E* × *B* shear layers during the repetition of the process without suffering from strong damping. It is also likely that the shear layers are accompanied by pressure corrugations since a part of the *E_r_* field originates from pressure corrugation through relaxation, while those due to zonal flows also cause pressure corrugation keeping the same in-phase relation as that due to the pressure relaxation. The *E* × *B* shear layers are found to propagate downward as discussed above.

It is also noted that spatial scales of processes 1 and 2 are different, which belongs to different regions in the size-PDF. However, both exhibit similar effective radial correlation lengths which belong to meso- and to macro-scale, indicating that both are responsible for globally constrained profile leading to an exponential function form with spatially constant scale length almost equal. Here, it is noted that process 2 provides a new pathway to extract a large amount of turbulent free energy from the system efficiently as seen in the size-PDF, which emerges when diamagnetic shear reflecting the global ion temperature profile is cancelled by that of the globally produced $E_{r}$ leading to $\theta_{0}∼0$. However, the level of cancellation depends on the relative amplitude between them, i.e. $\partial_{r}(\omega_{d}+\omega_{E})$, and is generally imperfect, which reduces the corresponding component, i.e. the region (c*). Furthermore, the cancellation takes place only for ion related mode so that $\omega_{d}$ has an opposite sign to $\omega_{E}$. Therefore, it is not expected in an electron mode such as TEM, which becomes important in higher electron temperature plasmas, and should be confirmed. However, even in this case, process 1, which has a similar effective radial correlation length, is considered to play a role in providing a constraint to the system. Furthermore, the radially extended global structures, which cause intermittent bursts, show a resemblance with the linear ITG modes, while it exceeds the radial width over linear mode estimated from the first-order ballooning mode theory. There is a case that the structure is not matched with the most unstable modes, but instead matched with secondary and/or tertiary unstable modes or even stable modes, which remains an unresolved problem.

An interesting concern is the variation of constraint with respect to the external power input. We observe the outer region to be less constrained for higher input power regime, which increases the core temperature despite the stronger constraint in the core region. As the physical mechanism to weaken the constraint in high input power regime, we considered the slower time scale evolution of *E* × *B* shear layers downward, which transfers the density perturbation across the knee to outer region and enhances the effective shearing rate there. The effect of input power on the dynamics of *E* × *B* shear layers is of specific importance and more detailed study for how micro-scale and/or meso-scale dynamics of *E* × *B* shear layers impact the global profile formation and relaxation.

## Data Availability

This article has no additional data.
